# Why “Heart-Failure?”

**Published:** 1892-06

**Authors:** 


### WHY "HEART-FAILURE"?

A prominent senator died in Washington the other day, "after a brief illness of only ten minutes." The newspaper report says, "his death was said by his physician to have been due to heart failure."

The New York *Herald* not long ago remarked: "It was heart failure," say the doctors, and they say it so often that we put on our thinking caps. One business man after another falls out of sight, and when we ask what the trouble was the reply is sure to be "heart failure!"

So it seems that, in addition to many well recognized and definite cardiac lesions, we must now include one other which, though vague and non-committal in its pathology, at least appears to be exceedingly common. When and how the term "heart-failure" originated we do not know, but we suspect that in some particular case of death the necessity for a diagnosis, conjoined with ignorance of the actual conditions present, had a great deal to do with it. We ease ourselves gracefully out of a difficulty by knowingly alleging "heart-failure" to be the cause of death in a particular instance. This is usually satisfactory to the friends of the deceased, but we are none the wiser thereby. A person dies nowadays without presenting very palpable and manifest reasons therefor, and it is fashionable for the laity nearly always, and the medical attendant too often, to say that "heart-failure" did it.

"Heart-failure" and "nervous prostration" are two unfortunate expressions in too common use. The latter is often used to cover a multitude of sins on the part of the patient and a hopeless ignorance on the part of his attendant. The two expressions we hear about as frequently, and are about as explicit as the prevalent "kidney trouble" and "womb disease." Nervous prostration, no doubt exists—sometimes, but that's neither here nor there. "Heart-failure," however, we believe, is neither a pathological condition nor a clinical fact.

As used at present the term would seem to mean the functional stoppage of the heart without sufficient assignable cause. If actual disease of the organ had been detected during life, then this vague and indefinite cause of death is no longer adduced. But why?

We believe that this notion is unscientific and contrary both to physiology and fact. It supposes that a normal heart may cease its functions without sufficient cause, intrinsic or extrinsic. Physiology, we think, will upold the statement that any organ will continue its healthy operation until something interferes to derange it, and this is as true of the lung and liver as it is of the heart. But we do not remember to have heard mention yet of "lung-failure." But why not? If the heart ceases to beat we believe there is a reason for it, and our failure before and after death to detect this reason surely does not warrant us in accusing this faithful organ of voluntarily (as it were) going on a strike and closing the scene. Actual heart-failure in the sense usually understood we do not believe exists, and the cases of death said to be so produced are due to other causes, whether these latter can be ascertained or not. Still when in our ignorance we assign this cause in a particular case it is generally accepted as ample by those who know no better.

We are glad to know that in some places the coroners and health departments no longer recognize "heart-failure" as a distinct entity. They will no longer accept this term as a sufficient cause of death, and the "physician who dares sign his certificates in such a manner is regarded as uncertain in his diagnosis and a stranger to modern pathology."

We have seen in one place a defence of the term with notes of a case. (See *Medical Mews*, No. 916.) A patient was taken in labor, and soon abundant hemorrhage occurred, with the passage of some clots; pulse 76, respiration normal. Cervix obliterated and os dilatable, head presenting. During two days profuse bleeding (sic) took place from time to time and several large clots were found about the os. Examination disclosed marginal placenta previa. Pulse now 72 and strong. Ordered one drachm extract ergot and tamponed the vagina. Labor now progressed, with pains of short duration. No further hemorrhage, but patient complained of faintness. Patient became hysterical. When head reached hollow of sacrum pains suddenly ceased, patient grew faint, pulse began to fail, and death supervened. At autopsy heart was found normal and "uterus contained no more blood than would normally appear after abstracting a child from a non-contracting cavity." There was an interstitial nephritis.

We do not see that this case can argue a great deal for the existence of "heart-failure" as a pathological entity. Why allege this as the cause of death when it appears that the patient had been losing blood profusely, from time to time, for as much as two days? The writer states that the actual amount of hemorrhage was not ascertained, but he supposed that it had been exaggerated. In this case we should say the heart "failed" because there was nothing left to contract upon.

We believe that if all such cases were carried to their last analysis, and all the data in a given case could be gathered in, "heart-failures" would be relegated to the shades of an obsolete and defunct pathology.

